# Blood-based gene expression signatures of medication-free outpatients with major depressive disorder: integrative genome-wide and candidate gene analyses

**DOI:** 10.1038/srep18776

**Published:** 2016-01-05

**Authors:** Hiroaki Hori, Daimei Sasayama, Toshiya Teraishi, Noriko Yamamoto, Seiji Nakamura, Miho Ota, Kotaro Hattori, Yoshiharu Kim, Teruhiko Higuchi, Hiroshi Kunugi

**Affiliations:** 1 Department of Mental Disorder Research, National Institute of Neuroscience, National Center of Neurology and Psychiatry, Tokyo, 187-8502, Japan; 2 Department of Adult Mental Health, National Institute of Mental Health, National Center of Neurology and Psychiatry, Tokyo, 187-8553, Japan; 3 DNA Chip Research Inc., Kanagawa, 230-0045, Japan; 4 National Center of Neurology and Psychiatry, Tokyo, 187-8502, Japan

## Abstract

Several microarray-based studies have investigated gene expression profiles in major depressive disorder (MDD), yet with highly variable findings. We examined blood-based genome-wide expression signatures of MDD, focusing on molecular pathways and networks underlying differentially expressed genes (DEGs) and behaviours of hypothesis-driven, evidence-based candidate genes for depression. Agilent human whole-genome arrays were used to measure gene expression in 14 medication-free outpatients with MDD who were at least moderately ill and 14 healthy controls matched pairwise for age and sex. After filtering, we compared expression of entire probes between patients and controls and identified DEGs. The DEGs were evaluated by pathway and network analyses. For the candidate gene analysis, we utilized 169 previously prioritized genes and examined their case-control separation efficiency and correlational co-expression network in patients relative to controls. The 317 screened DEGs mapped to a significantly over-represented pathway, the “synaptic transmission” pathway. The protein-protein interaction network was also significantly enriched, in which a number of key molecules for depression were included. The co-expression network of candidate genes was markedly disrupted in patients. This study provided evidence for an altered molecular network along with several key molecules in MDD and confirmed that the candidate genes are worthwhile targets for depression research.

Major depressive disorder (MDD) is a common psychiatric condition characterized by persistent low mood and/or diminished interest or pleasure in activities that used to be pleasurable. With an estimated 350 million people affected, depression is a leading cause of disability worldwide according to World Health Organization statistics. However, the pathogenesis is yet to be fully understood, and at present, there are no laboratory tests available to aid in its diagnosis or prognosis.

With the development of bioinformatics and systems biology methods, analysis of genome-wide gene expression patterns has emerged as a powerful tool to probe a molecular phenotype of a disease without requiring an *a priori* hypothesis. Using this technique, studies have suggested gene expression profiles as reliable markers for classification, prognostication and prediction of various diseases such as cancer^1^, autoimmune diseases^2^ and neurological disorders^3^. As gene expression profiling can reflect a dynamic picture of genomic responses to environmental contributions^4^, it is considered relevant to psychiatric disorders as well^5^,^6^,^7^,^8^.

Concerning the type of sample, earlier genome-wide expression studies in psychiatry often used post-mortem brain tissues as the target for sampling. Recently, much attention has turned to more easily accessible peripheral blood samples^8^,^9^. Several studies to compare gene expression in the blood and brain have demonstrated considerable overlap^10^,^11^, and as such, peripheral blood gene expression is regarded as a reasonable surrogate that circumvents several limitations associated with postmortem brain tissue^9^,^12^. Thus, blood gene expression profiling has become an important area of research in psychiatry^8^,^9^.

Accordingly, several microarray-based studies^13^,^14^,^15^,^16^,^17^,^18^,^19^ and one study using an RNA sequencing (RNA-seq) technique^20^ have investigated blood gene expression signatures in depressive disorders and identified a number of differentially expressed genes (DEGs). However, the reported DEGs have been highly variable across studies and, in general, most of the DEGs identified in one study have not been replicated in others^21^. This issue may have stemmed, at least in part, from different study design, psychotropic medications including antidepressants, and sample characteristics such as postpartum depression^13^, subsyndromal depression^16^, anxious depression^18^, and recurrent depression^20^. Alternatively, the stringent thresholds for a marker to be considered differentially expressed (that is, only those markers exceeding the thresholds adjusted for genome-wide error rate were selected as DEGs) may have contributed to the inconsistent findings. This explanation is plausible given the relatively small sample sizes and large numbers of transcripts simultaneously tested, and as a result, even true susceptibility genes for depression that do not necessarily have such a large effect size could be buried in a sea of false negative findings. Indeed, it is now widely accepted that depression is a multifactorial and heterogeneous disorder in which many susceptibility genes with small effects and a broad range of environmental factors, as well as the resultant complex gene-gene and gene-environment interactions, are likely to be in play^22^.

With respect to analysis and interpretation of genome-wide data, a growing interest has been directed toward pathways and networks that reflect dynamic interactions between genes, transcripts and proteins^23^, as they are considered to better harness the rich, comprehensive information in -omics data^24^,^25^. Assuming this, transcriptomic studies may benefit from searching for interactive relationships between expression levels of a relatively large number of genes that meet a less conservative statistical threshold. Such an approach may be especially advantageous to depression research^26^, given that the systems biology-oriented strategy could capture the complex disease process and, as a consequence, might give rise to the possibility of identifying (a set of) genes involved in depression.

On the other hand, genetic research for MDD in the past few decades has extensively studied the so-called candidate genes for depression that were primarily chosen based on their biological mechanisms, such as genes involved in monoamine and glutamate neurotransmission, hypothalamic-pituitary-adrenal (HPA) axis function, and neurogenesis. As there have been over 100 such genes implicated in depression^27^, genome-wide expression data will provide a unique opportunity to simultaneously examine these genes. Nonetheless, they have rarely been identified as DEGs in the above-mentioned transcriptome studies^13^,^14^,^15^,^16^,^17^,^18^,^19^,^20^, which may again be attributable to the rigorous threshold that could discard most of these genes with small to moderate sizes of effect. Hence, it would be of interest to test whether the hypothesis-driven, evidence-based candidate genes overall tend to be associated with depression, and, if so, which genes or gene networks are particularly relevant.

Against these backgrounds, the present study set out to investigate blood-based genome-wide expression signatures in patients with MDD relative to healthy controls matched pairwise for age and sex. The main aims were to examine how the identified DEGs would be translated into molecular pathways and networks and how the evidence-based candidate genes for depression would behave in the genome-wide expression data. We were particularly interested in inspecting the obtained results in the context of extant evidence in the literature.

## Results

**Figure 1:**
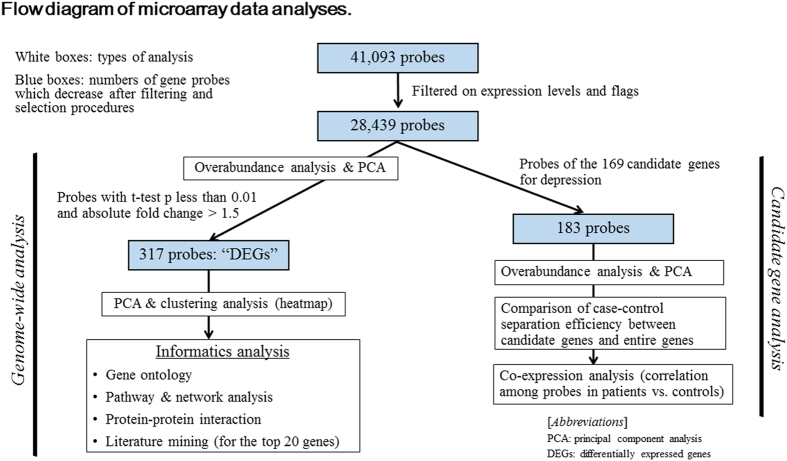
Flow diagram of microarray data analyses. The left arm of this figure represents steps of the genome-wide analysis and the right arm represents steps of the candidate gene analysis. White boxes indicate types of analysis. Blue boxes indicate numbers of gene probes, which decrease after filtering and selection procedures.

Characteristics of each sample are shown in Supplementary Table S1. The pipeline for microarray data analysis is illustrated in Fig. 1.

### Genome-wide analysis

The overabundance analysis of the filtered 28,439 probes illustrated that a greater number of differentially expressed probes between patients and controls were observed than would be expected by chance, across the entire p value range (Supplementary Fig. S1). The principal component analysis of the 28,439 probes showed that the two diagnostic groups were to some extent, but not sufficiently, linearly separable as either patient or control in the first two principal component dimensions (Supplementary Fig. S2).

Expression levels of the 28,439 probes were compared between patients and controls, using the hybrid thresholds of t-test p value less than 0.01 and absolute fold change greater than 1.5. This process yielded 317 DEGs, of which 247 were down-regulated and 70 were up-regulated in patients. The thresholding process is visualized as a volcano plot in Supplementary Fig. S3. Of the 317 DEGs, 234 probes with corresponding gene symbols are listed in Supplementary Table S2. A list of the top 20 DEGs is shown in Table 1; the top-hit gene was *LUZP1*, followed by *UGCG* and *UBQLN4*.

With respect to case-control separation performance of the DEGs, the principal component analysis of the 317 DEGs revealed that 24/28 (85.7%) of the samples were linearly separable (Supplementary Fig. S4). Results of the unsupervised hierarchical clustering on the 317 DEGs are displayed as a dendrogram in Supplementary Fig. S5, indicating that 23/28 samples (82.1%) were correctly classified.

**Figure 2:**
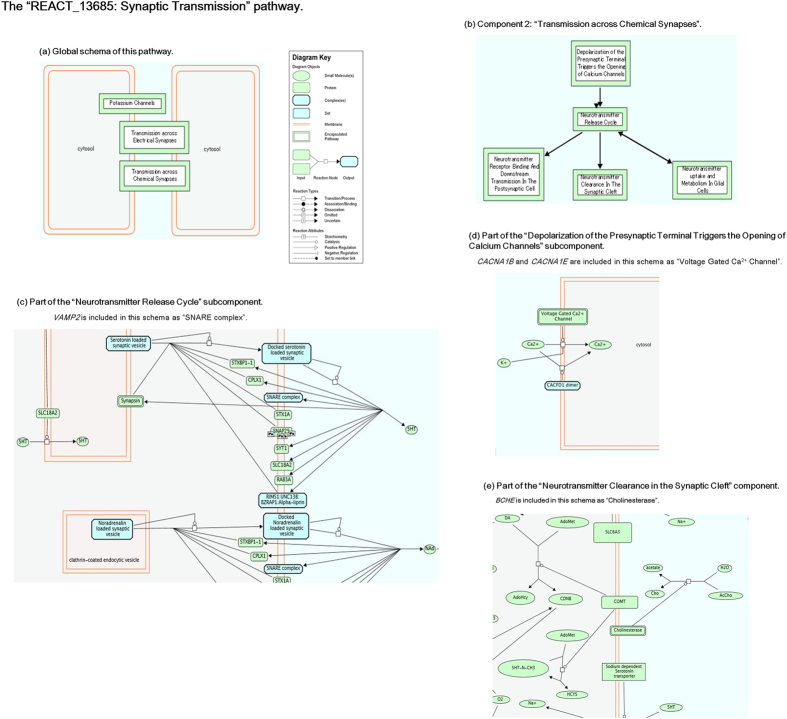
Schema of the “REACT_13685: Synaptic Transmission” pathway. To identify over-represented pathways, the DAVID software was used to map the input genes onto BioCarta, KEGG, and Reactome pathway databases, separately for the up-regulated (n = 52) and down-regulated (n = 182) genes. The only significant pathway was “REACT_13685: Synaptic Transmission” in the Reactome pathway database (p = 0.011) for the down-regulated genes. Four genes in the input gene list, ie. *BCHE*, *CACNA1B*, *CACNA1E*, and *VAMP2*, were included in this pathway. (**a**) Global schema of the pathway, showing that the pathway consists of 3 encapsulated pathway components: “Transmission across Electrical Synapses”, “Transmission across Chemical Synapses” and “Potassium Channels”. (**b**) Schema of the “Transmission across Chemical Synapses” component, in which all the four input genes are included. This schema shows that this component consists of 5 subcomponents. (**c**) Schema of a part of the “Neurotransmitter Release Cycle” subcomponent, in which *VAMP2* is included as “SNARE complex”. (**d**) Schema of a part of the “Depolarization of the Presynaptic Terminal Triggers the Opening of Calcium Channels” subcomponent, in which *CACNA1B* and *CACNA1E* are included as “Voltage Gated Ca^2+^ Channel”. (**e**) Schema of a part of the “Neurotransmitter Clearance in the Synaptic Cleft” subcomponent, in which *BCHE* is included as “Cholinesterase”.

**Figure 3:**
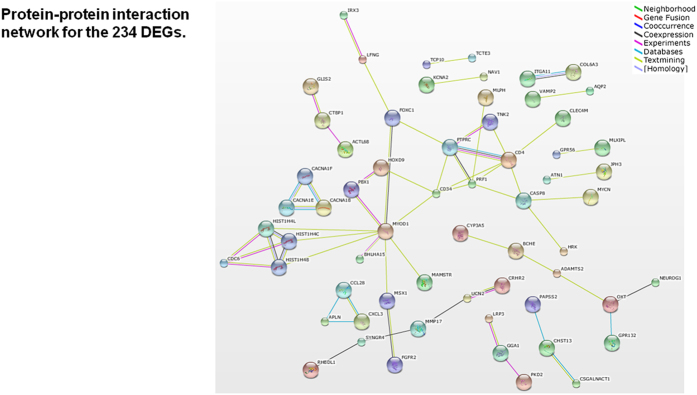
Protein-protein interactions between the DEGs as generated by the STRING database. Of the input 234 DEGs (ie. only those probes with corresponding gene symbols), 199 genes (proteins) matched the database and were used to construct the network. The depicted network includes all proteins (nodes) that had at least one interaction (edge) with other protein(s). Differently coloured edges indicate different types of evidence used in predicting interactions. Based on the statistical enrichment analysis incorporated in STRING, this entire network was significantly enriched (number of proteins used: 199; number of proteins observed: 62; number of proteins expected: 41.6; p = 0.00185).

Next we performed gene ontology (GO) and pathway/network analyses and conducted literature mining. The DAVID functional enrichment analysis revealed that there were 17 over-represented GO terms for the 182 down-regulated DEGs with gene symbols (Supplementary Table S3); in contrast, no terms were obtained for the 52 up-regulated DEGs. The 182 down-regulated DEGs and 52 up-regulated DEGs were separately mapped to the BioCarta, KEGG, and Reactome pathway databases, resulting in one significant pathway (p = 0.011) in the Reactome database, “REACT_13685: Synaptic Transmission”, for the down-regulated genes (Fig. 2a). Four genes in the input list, *BCHE*, *CACNA1B*, *CACNA1E* and *VAMP2*, were included in this pathway. This pathway consisted of 3 components: “Transmission across Electrical Synapses (Component 1)”, “Transmission across Chemical Synapses (Component 2)”, and “Potassium Channels (Component 3)”. All the 4 input genes were included in the Transmission across Chemical Synapses component (Fig. 2b–e) There were 280 genes (molecules) participating in the global pathway, of which 25 were found in the 169 candidate gene list of Kao *et al.*^27^ (Supplementary Table S4). Considering that the number of human genes listed in the leading repositories of genome annotation (eg. Ensembl, NCBI’s RefSeq and UCSC genome browser) is around 22,000^28^, the observed number was indicative of a remarkable over-representation (25/280 vs. 169/22,000; approximately 11.6 times greater). A protein-protein interaction network for the annotated 234 DEGs as determined by STRING is shown in Fig. 3. Of the input 234 genes, 199 genes (proteins) matched the database and were used to construct the network, of which 62 had at least one interaction with other protein(s) and constituted the entire network comprising several sub-networks. The statistical enrichment analysis incorporated in STRING revealed that the whole network was significantly enriched (number of proteins used: 199; number of proteins observed: 62; number of proteins expected: 41.6; p = 0.00185). This network consisted of a number of molecules previously reported to be associated with depression (according to a PubMed search), such as *CYP3A5*, *VAMP2*, *CSGALNACT1, CASP8*, *CRHR2*, *PKD2*, and *OXT* (Fig. 3). Results of literature mining for the top 20 DEGs using Chilibot and manual searches are presented in the right-side columns of Supplementary Table S5. Four genes, ie. *FKBP4*, *PKD2*, *CSGALNACT1*, and *VAMP2*, co-occurred with the term “depression”, and 11 genes were reported to be associated with brain function/structure.

The top 20 DEGs were compared with those reported in 6 previous transcriptome studies of MDD patients as compared to control subjects^14^,^15^,^17^,^18^,^19^,^20^ (Table 2). There were no genes listed as top-ranked DEGs in any two or more of these 7 studies.

### Candidate gene analysis

The overabundance analysis of the 183 probes showed that the number of differentially expressed candidate gene probes between patients and controls exceeded not only the number expected by chance but also the number observed using all 28,439 probes, albeit to a slight extent (Supplementary Fig. S6). Similarly, the principal component analysis revealed that the separation ability seemed to be somewhat greater (Supplementary Fig. S7a) compared to when using the entire probe (Supplementary Fig. S2). This ability was increased when only the top 11 genes that met hybrid thresholds of t-test p value less than 0.05 and absolute fold change greater than 1.5 were used, indicating that a relatively small number of candidate genes might be useful in separating patients from controls (Supplementary Fig. S7b).

When case-control separation efficiency was determined by a cut-off of absolute fold change greater than 1.5, 19 out of the 183 candidate gene probes exceeded the cutoff whereas 1,941 out of the 28,439 total probes did. Comparing these ratios (ie. 19/164 vs. 1,941/26,498) by chi-square test, the difference was nearly significant (p = 0.0575), suggesting a possible over-representation of candidate genes in depression. In contrast, when the cut-off was set using the t-test p value, the ability of candidate genes to discriminate patients from controls was almost the same as that of total genes (see Supplementary Fig. S6). Taken together, these results suggest that expression levels of the candidate genes were overall altered in depression, yet with marked variations within the patient group.

Of the candidate genes, *CRHR2* showed the largest difference in the expression levels between patients and controls; this was the only gene that exceeded the threshold of p less than 0.01 (Supplementary Table S6) and thus was included in the DEG list (Supplementary Table S2). In addition, *CRHR1* showed the third largest difference.

**Figure 4:**
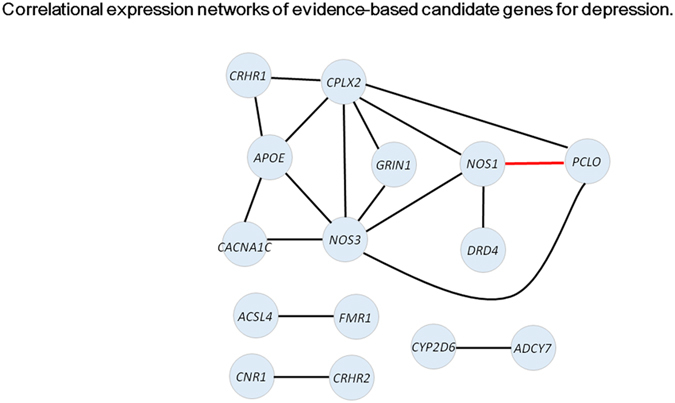
Correlational expression networks of evidence-based candidate genes for depression. Co-expression networks were examined separately for patients with MDD and healthy controls, using the 183 evidence-based candidate gene probes. Two genes were considered to be co-expressed if their Spearman’s rank order correlation was highly significant as defined by p < 1 × 10^−8^ (approximately corresponding to rho >0.97). There were 15 nodes (genes) and 18 edges (interactions) that exceeded this threshold (significant correlations between two probes representing the same gene are omitted). Edges in black indicate co-expression network in controls. The edge in red indicates common co-expression between patients and controls, which was the only co-expression exceeding the threshold in patients (that is, there were no co-expression networks specific to patients).

Correlational co-expression analyses among the 183 probes were performed separately for patients and controls, revealing that the numbers of significantly correlated probe pairs were 1,175 in patients and 2,602 in controls when the significance threshold was set at p < 0.01. When the threshold was set at p < 0.001, the numbers were 443 in patients and 1,243 in controls. These results indicated the disruption of the co-expression network in patients as compared to controls. Figure 4 presents only highly significant results where two gene probes were very strongly correlated with each other as defined by p < 1 × 10^−8^ (approximately corresponding to rho >0.97), which revealed remarkable disruption of the correlational co-expression network in patients (number of nodes: 2 vs. 15; number of edges: 1 vs. 18). The network for healthy subjects included two hub genes with 6 edges, namely *CPLX2* and *NOS3*. Two CRH receptor genes, *CRHR1* and *CRHR2*, were also found in this network.

## Discussion

In this study, we compared blood-based gene expression profiles between patients with MDD and matched healthy controls. We first conducted genome-wide analyses and then explored behaviours of hypothesis-driven candidate genes for depression. Our first main finding was that the 317 DEGs meeting the thresholds yielded a significantly enriched pathway and protein-protein interaction network. Importantly, this pathway and network were of considerable relevance to depression and to the central nervous system. Specifically, the significant over-representation of the synaptic transmission pathway agrees closely with the well-established evidence that impaired neurotransmission is a hallmark of depression and with recent findings of genome-wide significant signals within voltage-gated calcium channel genes (mainly *CACNA1C*) for major psychiatric disorders including MDD^29^,^30^. Moreover, the protein-protein interaction network included a number of molecules previously implicated in depression. Thus, the hypothesis that differential gene expression in depression can be better understood as a network of a relatively large number of genes/molecules was supported.

As described earlier, top-ranked DEGs in previous studies have been inconsistent, and the top 20 DEGs identified in the present study did not show any overlap with the previous findings (Table 2). These inconsistencies may be attributable to various factors such as ethnic differences, medications, and heterogeneity of depressive disorders. Nonetheless, a scrutiny of our top 20 DEGs through bioinformatics analyses including literature mining revealed several interesting findings. First, 11 of 20 genes have been shown to be involved in the brain. For example, the top-hit gene *LUZP1*^31^ and the third-hit *UBQLN4*^32^, albeit having no reported link with depression, have been demonstrated to be predominantly expressed in the brain, and the second-hit *UGCG* has been shown to be required in the maintenance of healthy intracellular interactions for nervous system stability and function^33^ (Supplementary Table S5). In addition, *FKBP4*, *PKD2*, *CSGALNACT1*, and *VAMP2* have been reported in relation to depression (or mood disorders). Of particular note here is that *VAMP2* and *CSGALNACT1* were both identified as the top-hit genes in genome-wide studies for depression^34^,^35^. VAMP (vesicle-associated membrane protein; synaptobrevin), together with syntaxin 1 and SNAP-25, forms the predominant neural SNARE (soluble N-ethylmaleimide-sensitive factor attachment protein receptor) complex that is primarily involved in vesicle fusion and plays essential roles in the regulation of voltage-gated calcium channels and neurotransmitter release. Here, we suggest that *VAMP2* is a key player in the pathogenesis of depression, based on the following convergent evidence. First, a microarray-based transcriptome study integrating animal and human data identified *VAMP2* as the only gene surpassing thresholds for all the different datasets, in which *VAMP2* was down-regulated^34^ (that study was not included in Table 2 because the primary transcriptome analysis was performed in mice). This finding is in striking agreement with the present result showing that this gene exhibited the greatest absolute fold change (down-regulation) in the top 20 DEGs (Table 1). Second, our pathway analysis revealed that *VAMP2* was a member of both the synaptic transmission pathway and protein-protein interaction network, suggesting an integral role that this gene plays in the molecular mechanism underlying depression. *CSGALNACT1* (chondroitin sulfate N-acetylgalactosaminyltransferase 1) is known to be involved in the synthesis of chondroitin sulfate, a major component of cartilage extracellular matrix. Interestingly, there is evidence that *CSGALNACT1* also has a role in brain development and neuroplasticity^36^. A genome-wide association study of antidepressant response revealed that *CSGALNACT1* harbours a variant most strongly associated with a sustained antidepressant response^35^. Furthermore, a genome-wide association study of nicotine dependence identified a genome-wide significant variant near this gene^37^. More recently, a reanalysis of microarray-based expression profiles in postmortem brain tissues from subjects with MDD identified upregulation of genes in the N-acetylgalactosaminyltransferase (GALNT) family (that is, genes known to be functionally related to *CSGALNACT1*^38^) as one of the most significant signals characterizing MDD^39^. These findings suggest a major involvement of *CSGALNACT1* in psychiatric disorders including depression. *FKBP4* and *PKD2* may also be of interest in light of the relevant literature. *FKBP4* (FK506 binding protein 4), together with *FKBP5*, participates in the regulation of the glucocorticoid receptor^40^ through which it possibly contributes to the pathophysiology of depression^41^. Regarding *PKD2*, there is one family study reporting co-segregation of mood disorders and autosomal-dominant polycystic kidney disease in which this gene plays a causal role^42^.

With respect to the performance of hypothesis-driven, evidence-based candidate genes for depression, the set of analyses to examine over-representation revealed that these genes overall tended to be associated with depression. The co-expression analyses showed that correlations among candidate genes were generally high for both diagnostic groups, supporting the putative small-world properties of the gene co-expression network^43^. However, in the present study this network was markedly disrupted in depression compared to that in controls. Relatedly, it has been shown that such a co-expression network in depression predicts subsequent treatment response^44^. *CPLX2* and *NOS3* were found to play central roles in the co-expression network for controls, and the result for *CPLX2* may be intriguing in the context of the finding for *VAMP2*. CPLXs (complexins) are small, SNARE-associated proteins that interact with the core SNARE complex to regulate calcium-triggered vesicle fusion^45^ (see Fig. 2c). In the present study, *CPLX2* was down-regulated in patients with the t-test p value less than 0.1 and with an approximately 2-fold change (Supplementary Table S5). Consistent with this observation, a mouse study showed that down-regulation of *CPLX2* leads to changes in neurotransmitter release that cause significant behavioural abnormalities mimicking depression^46^. Concerning the genes showing differential expression, the two CRH receptor genes, *CRHR2* and *CRHR1*, were the first and third strongest signals (respectively). The observed *lower* expression of *CRHR2* in depression is consistent with a microarray study in rats in which down-regulation of *CRHR2* was observed after chronic immobilization stress^47^. Furthermore, *CRHR2* and *CRHR1* participated in the co-expression network for healthy controls only, indicating their decreased connectivity with other genes in depression (Fig. 4). These results agree with the well-documented evidence for the pivotal role of HPA axis dysfunction in the pathophysiology of depression. Our results also suggest that expression levels of candidate genes vary markedly within the patient sample, potentially pointing to the existence of different subtypes of MDD. In line with this, there are several studies that have successfully subtyped depression based on microarray-based genome-wide gene expression profiles^16^,^34^,^48^. Such an approach might lead to more sophisticated diagnosis of MDD and ultimately open a new avenue for tailor-made treatment strategies for each patient. Taken together, our results indicate that candidate genes for depression—which, if excessively stringent statistical thresholds are used, will be mostly discarded due to their moderate effect sizes (as was the case with the present study) —are indeed a promising target for depression research.

The current study has several limitations. Firstly, confirmation of the present results was not performed using an independent replication sample, which means that some of the identified DEGs may merely represent false-positive results. This absence of confirmation data is due to the difficulty of sampling non-medicated MDD patients with at least moderate depression severity. Still, it may be worth noting that this study itself had a confirmatory nature, in that we inspected the obtained results using multiple lines of evidence and, most notably, replicated the associations of depression with genome-wide supported *VAMP2* and *CSGALNACT1* as well as the evidence-based candidate genes. Perhaps relatedly, our sample was well defined since the MDD patients were all outpatients, medication-free, at least moderately ill, and matched pairwise with control subjects for age and sex. Secondly, this study was cross-sectional, which precluded inferences about whether the differential expression pattern observed in patients represents a preexisting vulnerability or is a consequence of depressive illness. Thirdly, we should mention that methodological advances in sequencing and bioinformatics have enabled next-generation whole-transcriptome sequencing technology, ie. RNA-seq. Still, microarray data have been demonstrated to show high concordance with RNA-seq data^49^, particularly when weakly expressed genes are excluded^50^. As described below, we utilized the Agilent microarray platform with a wide dynamic range of 5 orders of magnitude, and moreover, filtered out those genes with low expression values.

In summary, the present microarray-based gene expression profiling for depression provided evidence for an altered molecular network along with key molecules and supported that the candidate genes are worthwhile targets for depression research. Findings from the present and previous studies converge on several genes considered to play integral roles in depression, particularly *VAMP2, CSGALNACT1*, and *CRHR2*, which might be followed by *LUZP1*, *UGCG*, *UBQLN4*, *FKBP4*, *PKD2*, *CPLX2*, and *CRHR1*. Future well-designed transcriptome studies, integrated functional genomics studies^51^, and reanalysis/meta-analysis of expression data deposited in the public domain^52^ should help to further understand gene expression signatures of depression and to establish genes useful in its prediction, diagnosis and treatment.

## Methods

### Participants

Supplementary Table S1 shows the characteristics of each sample. The sample size was determined based on previous microarray-based genome-wide expression studies on depression^13^,^14^,^15^,^16^,^17^.

Fourteen outpatients with current MDD (mean age 41.6 [standard deviation 11.3] years, ranging from 26 to 57 years; 7 women) were recruited from the outpatient clinic of the National Center of Neurology and Psychiatry (NCNP) Hospital, Tokyo, Japan, or through an advertisement in a free local magazine and our website announcement. Consensus diagnoses were based on clinical interviews, observations and case notes by at least two experienced psychiatrists. The clinical diagnosis was confirmed using either the Structured Clinical Interview for DSM-IV Axis-I disorders^53^ or the Mini-International Neuropsychiatric Interview (MINI)^54^ by a trained research psychiatrist. All patients were free of psychotropic medication for at least 1 month at the time of blood sampling. Only those patients whose depression severity was at least moderate, as defined by the total score on the Hamilton Depression Rating Scale 21-item version (HAM-D)^55^ of 18 or more^56^, were enrolled (mean HAM-D total score 25.2 [standard deviation 5.8], ranging from 18 to 37; Supplementary Table S1). Patients with bipolar disorders were excluded.

Fourteen healthy control participants, matched pairwise for sex and age (age difference within a given matched pair needed to be 3 years or less), were recruited from the same geographical area via a free local magazine and website announcement (Supplementary Table S1). They were interviewed using the MINI by a research psychiatrist, and only those without current Axis-I psychiatric disorders were enrolled. A non-structured interview was also conducted by an experienced psychiatrist to exclude individuals who demonstrated one or more of the following conditions: having past or current regular contact to psychiatric services, having a history of regular use of psychotropics, and presenting other obvious self-reported signs of past primary psychotic and mood disorders.

Additional common exclusion criteria were having a history of central nervous system disease, severe head injury and substance abuse/dependence. The present study was approved by the ethics committee of the NCNP, Tokyo, Japan, and was conducted in accordance with the Declaration of Helsinki. After description of the study, written informed consent was obtained from every participant.

### mRNA extraction and processing

Venous blood was collected in PAXgene Blood RNA Tubes (Becton, Dickinson and Company, Tokyo Japan) between 11 a.m. and 12 a.m. from each participant and was incubated at room temperature for 24 hours for RNA stabilization. RNA was extracted from whole blood according to the manufacturer’s guidelines using the PAXgene Blood RNA System Kit (Qiagen, Tokyo, Japan). Quality and quantity of the RNA samples were determined using a NanoDrop ND-1000 spectrophotometer (NanoDrop Technologies, Inc., Wilmington, DE, USA) and an Agilent 2100 Bioanalyzer (Agilent Technologies, Tokyo, Japan).

Gene expression analysis was performed on Agilent Whole Human Genome 4 × 44 K arrays (Agilent Technologies). Cy3-labelled cRNA was synthesized from 250 ng of total RNA using an Agilent Quick Amp Labeling Kit (Agilent Technologies). The Agilent one-color microarray platform is demonstrated to have high (approximately 90%) concordance with TaqMan qPCR assays^57^. Raw signal data were analysed using the GeneSpring GX software, version 13.0 (Agilent Technologies). The raw signal values were thresholded to 1.0, and log base 2-transformation was performed. The 75th percentile shift normalization and baseline transformation with the median of all samples were then conducted, as per recommendations in the GeneSpring manual. The normalized data were used for all analyses. To enhance reproducibility of measurements, quality control was applied by an expression filter with 20th percentile threshold to remove genes with low signal intensity values, followed by filtering on flags based on the default settings. These steps resulted in 28,439 probes, for which all subsequent analyses were performed (Fig. 1).

### Microarray data analysis: genome-wide analysis

Steps of the data-driven genome-wide gene expression analysis are shown in the left arm of Fig. 1. The 28,439 probes after filtering were first evaluated by overabundance and principal component analyses in order to assess the performance of the entire expression dataset with respect to the separation of the samples into patients and controls. Next we compared expression levels of these probes between patients and controls and determined DEGs by hybrid thresholds of p-value for moderated t-test less than 0.01 combined with absolute fold change greater than 1.5. In this analysis, conservative correction methods for multiple testing such as false discovery rate were left out because they were found to be too stringent for the present purpose of selecting a relatively large number (eg. some hundreds) of DEGs with which to make subsequent analyses more pertinent. The identified DEGs were then used for all downstream analyses unless otherwise specified.

To assess case-control separation efficiency of the DEGs, principal component analysis was performed. Unsupervised hierarchical clustering analysis, with Euclidean distance as a measure of similarity coupled with the average linkage method, was applied to stratify samples based on expression levels of the DEGs, as this algorithm has been shown to produce relatively stable and accurate clusters^58^. The principal component analysis and clustering analysis were performed using the GeneSpring software. The DEGs were then evaluated by a sequence of bioinformatics analyses comprising GO analysis, pathway/network analysis, protein-protein interaction, and literature mining (see Supplementary Method for details). These informatics analyses were conducted using publicly available databases. To understand the biological significance and evaluate the statistical enrichment (over-representation) of the DEGs, we first conducted GO term enrichment analysis using the Database for Annotation, Visualization and Integrated Discovery (DAVID) v6.7 functional enrichment tool^59^. DAVID was then used to identify over-represented pathways. Specifically, the DEGs uploaded to DAVID were mapped onto BioCarta, Kyoto Encyclopedia of Genes and Genomes (KEGG), and Reactome pathway databases, separately for the up-regulated and down-regulated genes. To construct a protein-protein interaction network and analyse its enrichment, the DEGs were submitted to the Search Tool for the Retrieval of Interacting Genes/Proteins (STRING) v9.1 software^60^. Finally, literature mining was performed to scrutinize the top 20 genes of the DEG list in terms of their relevance to depression and the central nervous system. To this end, a literature analysis software was used to identify co-occurrence of names of a given gene (or genes) and a given keyword. The search term “depression” or “central nervous system” was used as the keyword. In addition, manual PubMed literature searches were conducted on the same 20 genes to check for other important evidence for their involvement in the brain.

### Microarray data analysis: evidence-based candidate gene analysis

For the candidate gene analysis, we decided to include 169 genes as depression candidate genes, based on the study of Kao *et al.*^27^. In that study, candidate genes for MDD were prioritized using multiple lines of evidence-based datasets consisting of association, linkage, gene expression (including both human and animal studies), regulatory pathway, and literature searches. The number of genes (ie. 169) as well as the content of this list was therefore considered adequate for the present study.

The procedure for the candidate gene analysis is illustrated in the right arm of Fig. 1. Of the 169 genes, 113 genes corresponding to 183 probes survived the initial filters of expression levels and flag status. Similar to the genome-wide analysis, overabundance and principal component analyses were used to assess the performance of the 183 probes.

To test if the correlational co-expression network among candidate genes is disrupted in MDD, correlations between expression levels of the 183 probes (ie. _183_C_2_ = 16,653 combinations) were assessed in each diagnostic group by the Spearman’s rank order correlation using IBM SPSS Statistics version 21.0 (IBM Corp., Tokyo, Japan). The nonparametric analysis was used because assumptions of normality as determined by the Shapiro-Wilk test were violated in 26 of the 183 probes in at least one diagnostic group (ie. patients or controls).

## Additional Information

**How to cite this article**: Hori, H. *et al.* Blood-based gene expression signatures of medication-free outpatients with major depressive disorder: integrative genome-wide and candidate gene analyses. *Sci. Rep.* **6**, 18776; doi: 10.1038/srep18776 (2016).

## Supplementary Material

Supplementary Information

Supplementary Dataset 1

## Figures and Tables

**Table 1:** Top 20 differentially expressed genes in depressed patients compared with controls.

Probe name	GeneSymbol	p	Fold change	Description	EntrezGene ID	RefSeq accession number
A_23_P407142	*LUZP1*	8.44E-05	−1.98	leucine zipper protein 1, transcript variant 1	7798	NM_033631
A_23_P123645	*UGCG*	2.16E-04	1.59	UDP-glucose ceramide glucosyltransferase	7357	XM_005252186
A_23_P201059	*UBQLN4*	2.40E-04	−1.91	ubiquilin 4	56893	NM_020131
A_32_P902957	*ARL11*	4.29E-04	1.51	ADP-ribosylation factor-like 11	115761	NM_138450
A_24_P257201	*MRPL30*	4.83E-04	1.58	mitochondrial ribosomal protein L30, transcript variant 1	51263	NM_145212
A_23_P214944	*ALDH8A1*	6.65E-04	1.89	aldehyde dehydrogenase 8 family, member A1, transcript variant 1	64577	NM_022568
A_23_P128372	*FKBP4*	7.11E-04	−1.99	FK506 binding protein 4, 59kDa	2288	NM_002014
A_23_P40785	*ZNF445*	7.82E-04	−2.07	zinc finger protein 445	353274	NM_181489
A_23_P211619	*SCUBE1*	7.84E-04	−1.67	signal peptide, CUB domain, EGF-like 1	80274	NM_173050
A_23_P167324	*PKD2*	7.94E-04	1.88	polycystic kidney disease 2 (autosomal dominant)	5311	NM_000297
A_24_P406525	*CSGALNACT1*	8.66E-04	1.71	chondroitin sulfate N-acetylgalactosaminyltransferase 1, transcript variant 2	55790	NM_018371
A_23_P65320	*NEK3*	0.00103	1.68	NIMA-related kinase 3, transcript variant 1	4752	NM_002498
A_32_P209148	*CCDC41-AS1*	0.00104	−1.75	CCDC41 antisense RNA 1 (head to head), long non-coding RNA	144486	NR_027035
A_23_P206284	*GPR56*	0.00107	−1.64	G protein-coupled receptor 56, transcript variant 3	9289	NM_201525
A_23_P158349	*RABL3*	0.00108	1.51	RAB, member of RAS oncogene family-like 3	285282	NM_173825
A_24_P166407	*HIST1H4B*	0.00112	1.84	histone cluster 1, H4b	8366	NM_003544
A_24_P123768	*EIF2AK1*	0.00115	−1.86	eukaryotic translation initiation factor 2-alpha kinase 1, transcript variant 1	27102	NM_014413
A_23_P328735	*THAP6*	0.00117	1.80	THAP domain containing 6	152815	NM_144721
A_23_P119857	*TTC32*	0.00122	1.64	tetratricopeptide repeat domain 32	130502	NM_001008237
A_24_P313576	*VAMP2*	0.00126	−2.22	vesicle-associated membrane protein 2 (synaptobrevin 2)	6844	NM_014232

Sorted by p value for the t-test in an ascending order. Probes without corresponding gene symbols are excluded.

Positive and negative fold change values represent up-regulated and down-regulated expressions in depression, respectively.

**Table 2:** Comparisons of top-ranked differentially expressed genes between blood-based gene expression profiling studies for MDD.

Present study^a^	Jansen *et al*., 2015	Guilloux *et al*., 2015	Mostafavi *et al*., 2014	Liu *et al*., 2014^a^	Belzeaux *et al*., 2012^a^	Menke *et al*., 2012^b^
*LUZP1* ↓	*KLRD1* ↓	*KRT1* ↑	*MINOS1* ↓	*SND1-IT1* ↑	*CELF2* ↑	*GPR114* ↓
*UGCG* ↑	*IL2RB* ↓	*NA* ↑	*COPG* ↑	*Q9P1I9* ↑	*MAT2A* ↑	*ICA1* ↑
*UBQLN4* ↓	*GZMB* ↓	*MS4A7* ↓	*SYF2* ↓	*LOC728093* ↑	*CASKIN1* ↓	*NCBP1* ↓
*ARL11* ↑	*LRRC4* ↑	*AFAP1* ↑	*EHMT2* ↑	*Q9P194* ↑	*SNORA28* ↓	*P2RY2* ↓
*MRPL30* ↑	*CPEB4* ↑	*UBB* ↑	*UBAP2L* ↑	*LOC100124692* ↑	*POTEE* ↑	*TPST1* ↑
*ALDH8A1* ↑	*CALM1* ↓	*GLRX5* ↑	*MAFK* ↓	*Q6ZNT7* ↑	*ARHGEF6* ↑	
*FKBP4* ↓	*TGFBR3* ↓	*KYNU* ↓	*MX1* ↑	*FNDC9* ↑	*TBC1D5* ↑	
*ZNF445* ↓	*PVRL1* ↑	*PIK3CD* ↑	*C1ORF101* ↑	*OR52K2* ↑	*ZFAND3* ↑	
*SCUBE1* ↓	*SNRPD3* ↓	*PNN* ↓	*SNRNP35* ↓	*C10orf31* ↑	*API5* ↑	
*PKD2* ↑	*APOBEC3G* ↓	*FLOT1* ↑	*FAM120AOS* ↓	*Q9UI72* ↑	*LOC391334* ↑	
*CSGALNACT1* ↑	*PTPN4* ↓	*NA* ↓	*NOP2* ↑	*GAFA2* ↑	*XRN2* ↑	
*NEK3* ↑	*KLRK1* ↓	*C2orf47* ↓	*SRSF5* ↓	*C9orf84* ↑	*ACTBL2* ↑	
*CCDC41-AS1* ↓	*DYSF* ↑	*AGAP10* ↑	*TNFRSF10B* ↑	*AIM2* ↑	*MINPP1* ↑	
*GPR56* ↓	*TNFRSF10C* ↑	*HDAC7* ↑	*PIPOX* ↑	*OR52K1* ↑	*POTEF* ↑	
*RABL3* ↑	*NCALD* ↓	*SYT15* ↑	*OAS1* ↑	*Q9P1M3* ↑	*OPA3* ↑	
*HIST1H4B* ↑	*C14orf1* ↓	*NA* ↓	*RABEPK* ↑	*IFNK* ↑	*ADIPOR1* ↑	
*EIF2AK1* ↓	*COX18* ↓	*KIAA1731* ↓	*SEMA3C* ↑	*Q9P1I3* ↑	*OSBPL9* ↑	
*THAP6* ↑	*SSH2* ↑	*COMMD1* ↓	*CINP* ↓	*KY* ↑	*CTBP1* ↑	
*TTC32* ↑		*GRAMD1A* ↑	*H6PD* ↓	*Q6UXQ5* ↑	*BX095413* ↓	
*VAMP2* ↓		*DNAJC13* ↑	*CCNI* ↓	*Q6ZNS0* ↑	*GSPT1* ↑	

Sorted by p value in an ascending order, except for the study of Menke *et al.* (2012). Probes without corresponding gene symbols are excluded.

Upward and downward arrows represent up-regulated and down-regulated expressions in depression, respectively.

*Abbreviation:* MDD, major depressive disorder.

The studies of Segman *et al.* (2010) and Yi *et al.* (2012) are not included in this table; In Segman *et al.* (2010), statistics such as p value and fold change were not provided in their differentially expressed gene list; In Yi *et al.* (2012), data on direct comparisons between MDD patients and healthy controls were not provided.

^a^Probes with absolute fold change < 1.5 were excluded.

^b^Differentially expressed genes with p < 0.05 and absolute fold change >1.15 in two independent sample sets were listed.
