# Supplementary material for: Blood-based gene expression signatures of medication-free outpatients with major depressive disorder: integrative genome-wide and candidate gene analyses
Source: Sci Rep. 2016 Jan 5;6:18776. doi: 10.1038/srep18776 (PMC4700430; doi:10.1038/srep18776)
Supplement: Supplementary Information [file srep18776-s1.pdf]

Blood-based gene expression signatures of medication-free outpatients with major depressive disorder: integrative genome-wide and candidate gene analyses

Hiroaki Hori[1,2], Daimei Sasayama[1], Toshiya Teraishi[1], Noriko Yamamoto[1], Seiji Nakamura[3], Miho Ota[1], Kotaro Hattori[1], Yoshiharu Kim[2], Teruhiko Higuchi[4] & Hiroshi Kunugi[1]

[1] Department of Mental Disorder Research, National Institute of Neuroscience, National Center of Neurology and Psychiatry, Tokyo, 187-8502, Japan
[2] Department of Adult Mental Health, National Institute of Mental Health, National Center of Neurology and Psychiatry, Tokyo, 187-8553, Japan
[3] DNA Chip Research Inc., Kanagawa, 230-0045, Japan
[4] National Center of Neurology and Psychiatry, Tokyo, 187-8502, Japan

Supplementary material 1: Supplementary Figures & Supplementary Method

Supplementary Figure S1

Overabundance plot showing the number of gene probes (among the total 28,439 probes) that were differentially expressed between patients and controls as determined by the moderated t-test.

(a) Entire range of p value.

(b) Enlarged view of the bottom left region (ie. the range of p less than 0.05) indicated by the dotted lines in (a).

These figures illustrate that the number of observed differences (solid line) exceeded the number of differences expected by chance (dotted line) across the entire p value range.

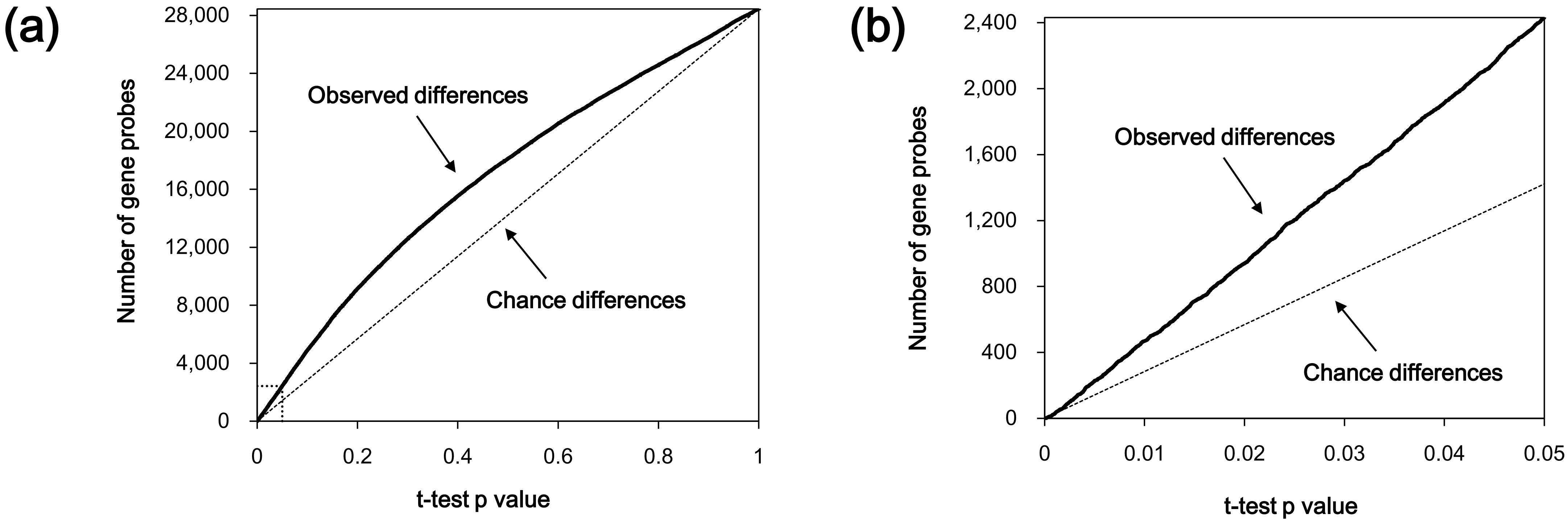

Supplementary Figure S2

Principal component analysis plots for expression profiles of the total 28 subjects, using the filtered 28,439 probes.

The first two principal components are presented.

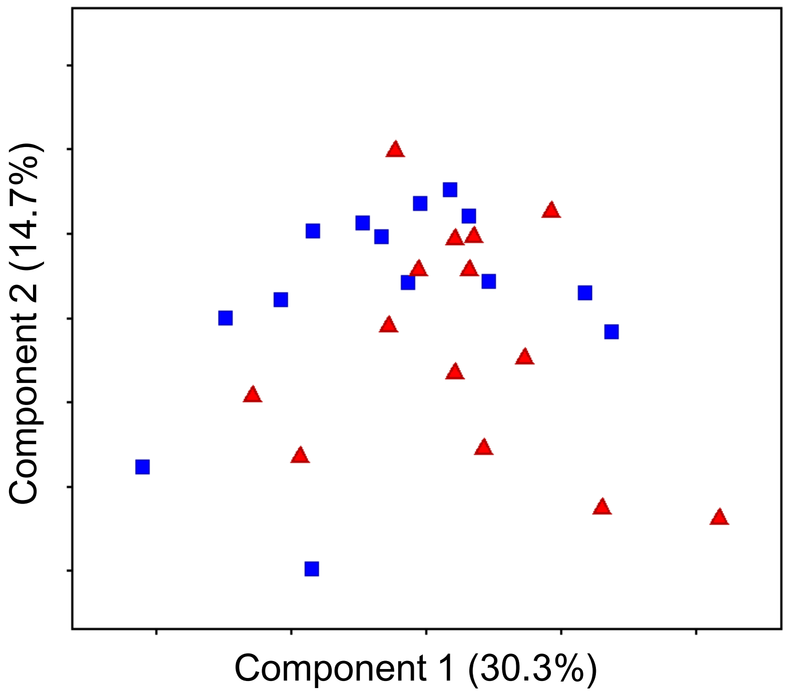

Red triangle: Depressed patient;
Blue square: Healthy control subject

The two diagnostic groups were to some extent, but not sufficiently, linearly separable as either patient or control in the first two principal component dimensions.

Supplementary Figure S3

Volcano plot of differentially expressed genes between patients and controls.

The x-axis represents the logarithm base 2 of the fold change of expression in patients relative to controls. The y-axis represents the negative logarithm base 10 of the p value for the moderated t-test comparing expression between patients and controls. Gene probes located in the right arm of the volcano are up-regulated in depression and those located in the left arm are down-regulated.

Vertical green lines indicate the thresholds of fold change (i.e., 1.5 and 1/1.5). A horizontal green line indicates the significance threshold of p value (i.e., 0.01). Thus defined, blue dots in the upper-right and upper-left corners represent those gene probes (n = 317) that passed the hybrid thresholds, and the remaining grey dots represent those probes that did not pass one or both of the thresholds.

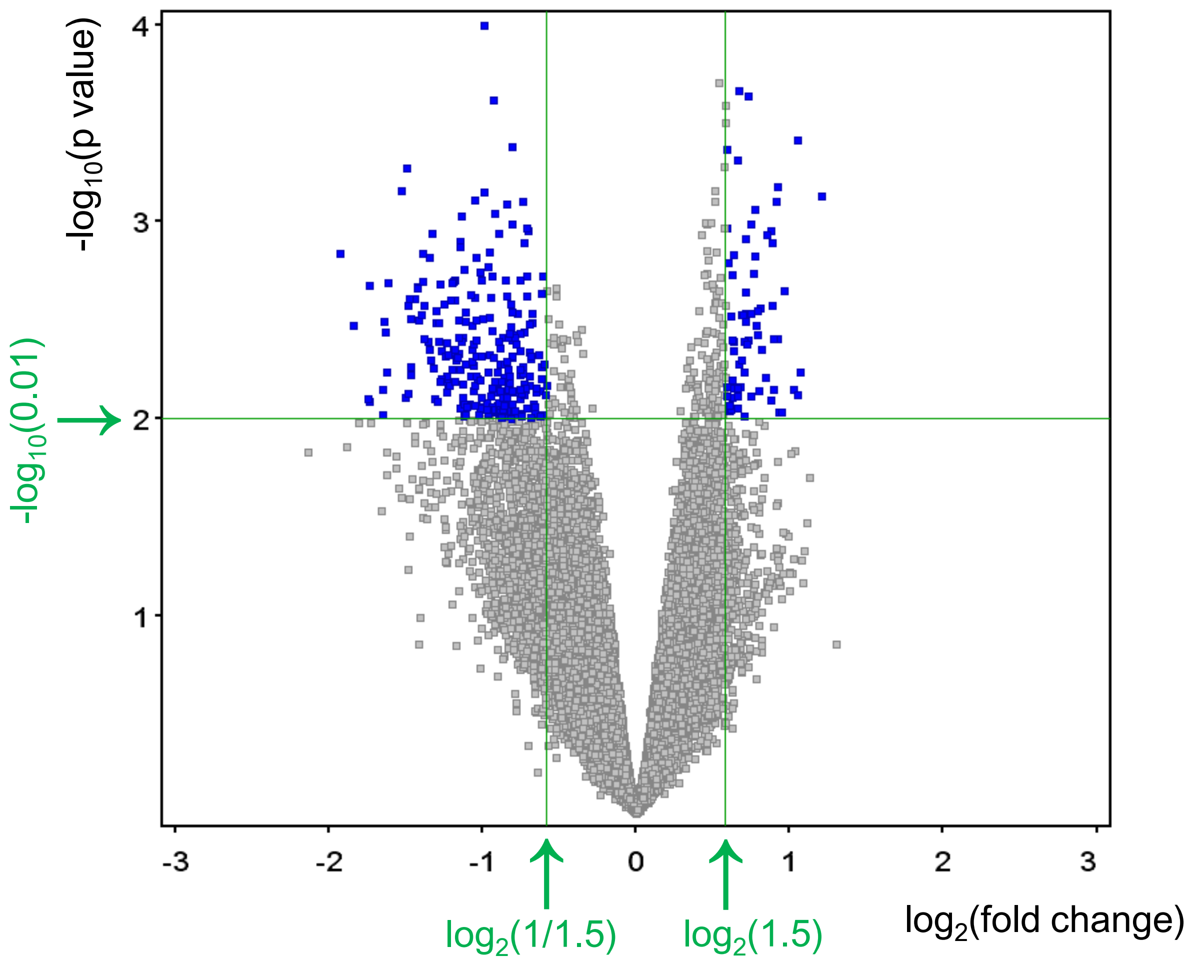

Supplementary Figure S4

Principal component analysis plots for expression profiles of the total 28 subjects, using the 317 probes with p less than 0.01 and absolute fold change greater than 1.5.

The first two principal components are presented.

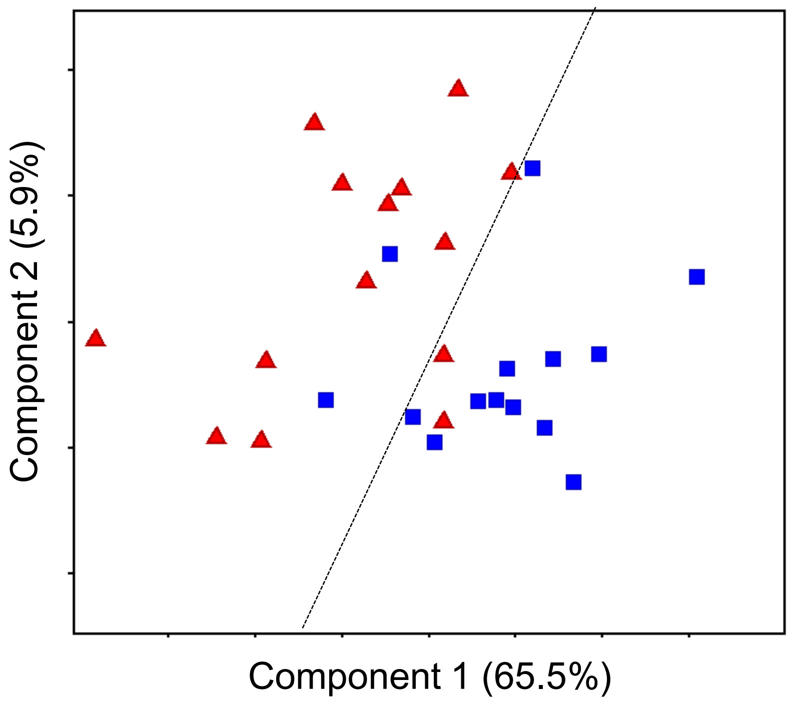

Red triangle: Depressed patient;
Blue square: Healthy control subject

As expected, separation efficiency was increased compared to that using the entire probes (Figure S2).

Supplementary Figure S5

Unsupervised hierarchical clustering of patients and matched controls using the 317 differentially expressed gene probes.

The dendrogram was created by the clustering procedure described below. Sample ID was defined in Supplementary Table S1. The bottom panel shows the expression values for each sample by each probe arranged vertically.

Distances between samples were determined by the Euclidean distance as a measure of similarity combined with the average linkage method.

Four patients with overall low expression patterns were first isolated, followed by one control subject with overall high expression pattern. The remaining 23 samples were subdivided into two large clusters, with the left one consisting mostly of controls and the right one patients. One patient and four control subjects were misclassified, resulting in 23/28 (82.1%) correctly classified samples.

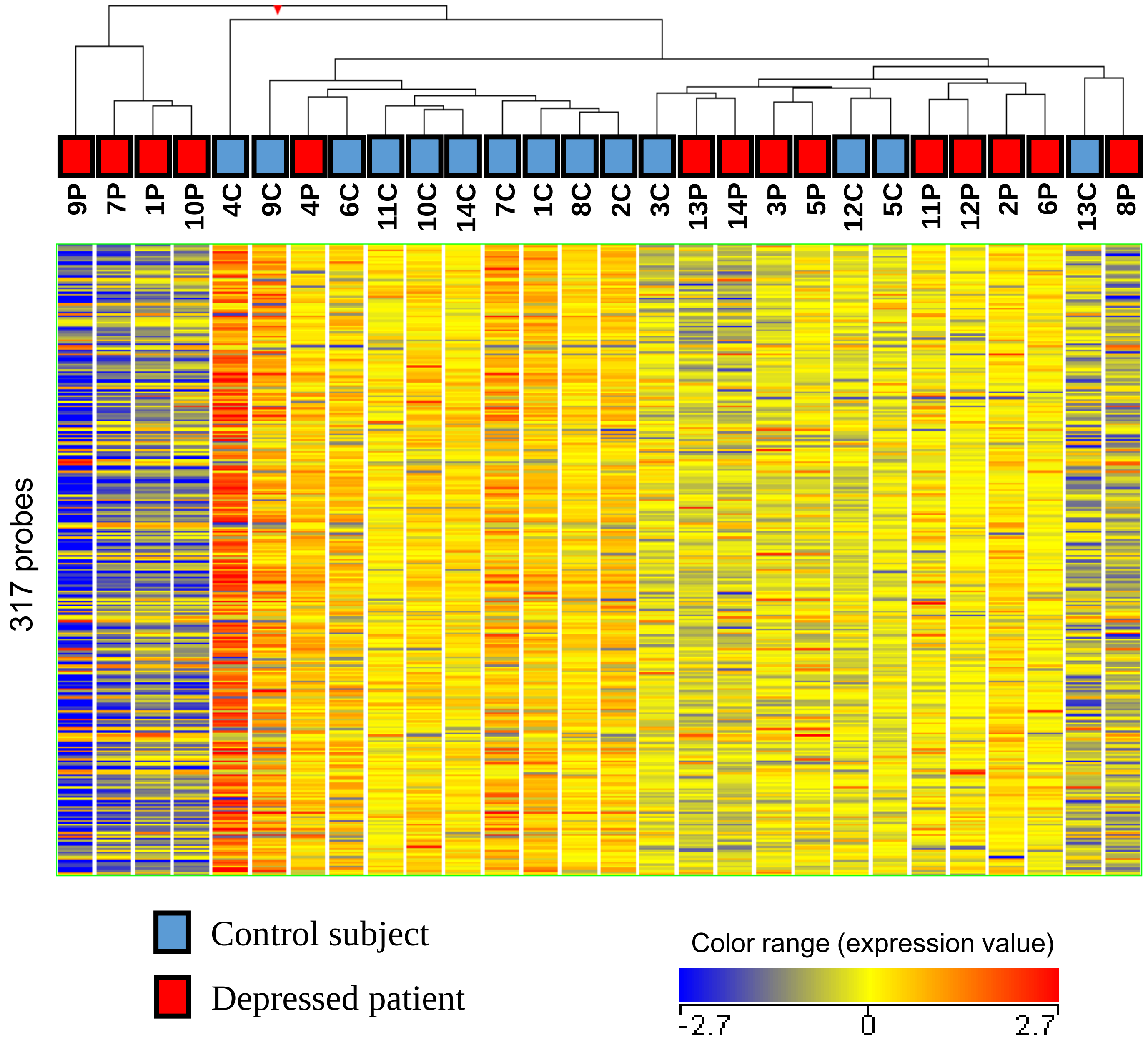

Supplementary Figure S6

Overabundance plot showing the number of gene probes (among the 183 candidate gene probes) that were differentially expressed between patients and controls as determined by the moderated t-test.

The thick bumpy line comprises each of the 183 probes.
The dotted straight line indicates the chance difference level.
The solid curve is the superimposed line of Figure S1a (only the shape is retained, with the vertical axis scale being irrelevant to this curve).

This figure indicates that the number of observed differences in candidate gene probes (bumpy line) exceeded not only the number of differences expected by chance (dotted line) but also the number of differences using the total probe (curved line) albeit to a slight extent.

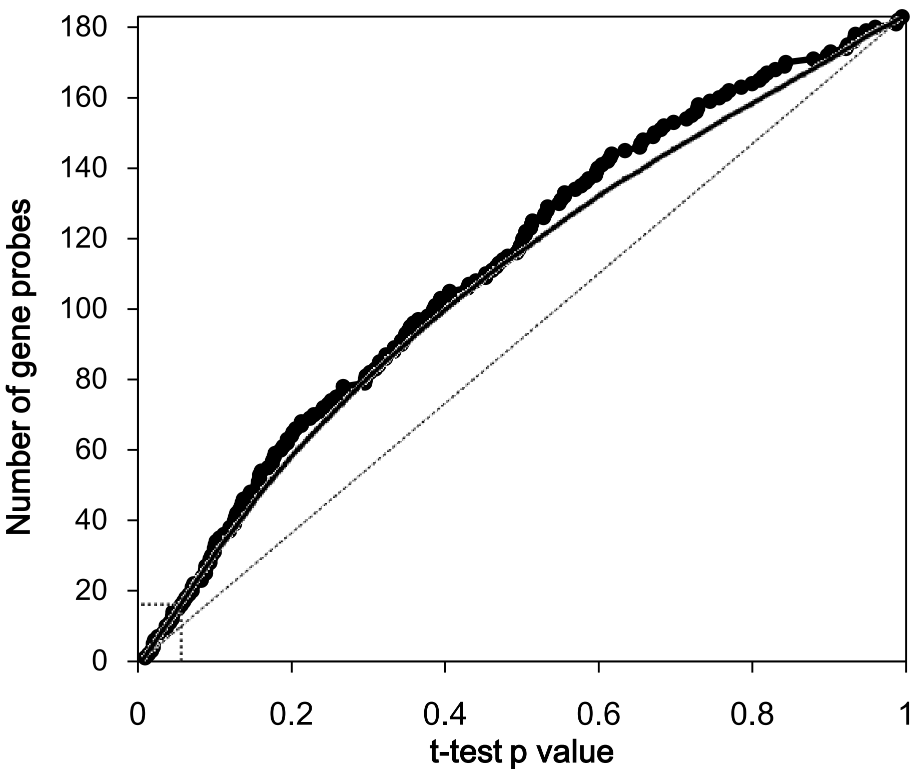

Supplementary Figure S7

Principal component analysis plots for expression profiles of the total 28 subjects, using the candidate gene probes for depression.

The first two principal components are presented.

(a) Using the 183 candidate gene probes.

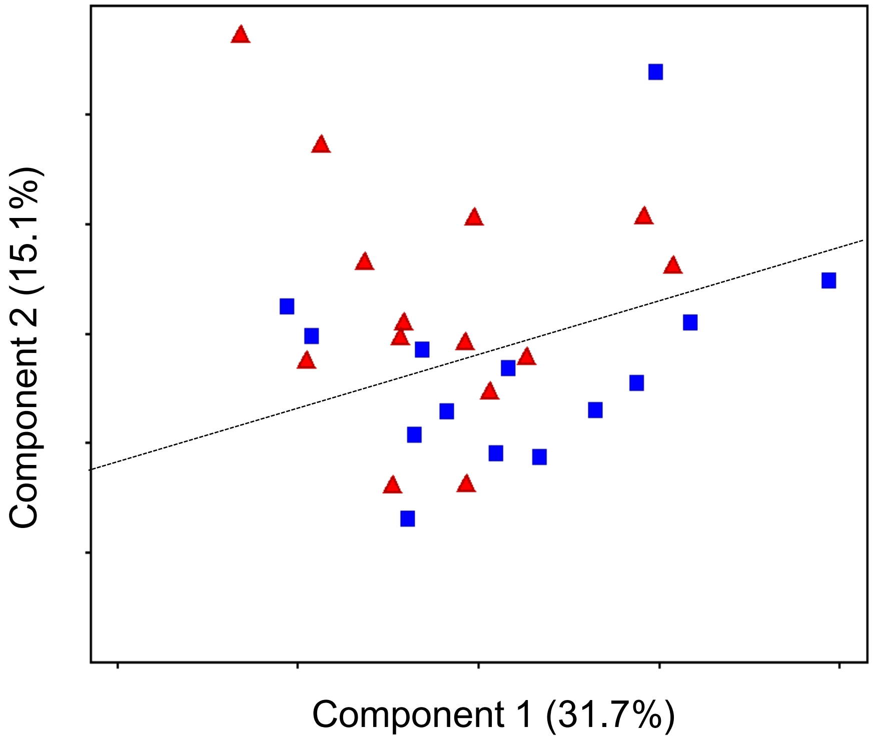

Separation efficiency was somewhat greater than when using the entire probes (Figure S2).

(b) Using the 11 probes surpassing hybrid thresholds of p less than 0.05 and absolute fold change greater than 1.5.

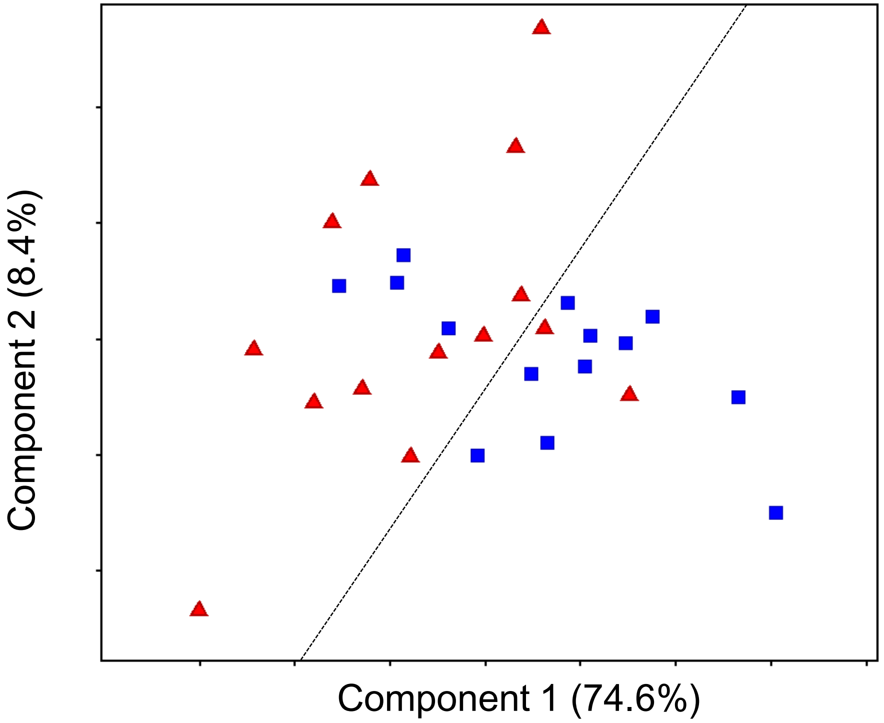

Separation efficiency was increased, suggesting that relatively small number of candidate genes may be useful in discriminating depressed patients from controls.

Red triangle: Depressed patient;
Blue square: Healthy control subject

Supplementary Method

Microarray data analysis: genome-wide analysis *Bioinformatics analysis*

The identified differentially expressed genes (DEGs) were subjected to a sequence of bioinformatics analyses comprising gene ontology, pathway/network analysis, protein-protein interaction and literature mining. These informatics analyses were conducted using publicly available databases. To understand the biological significance and evaluate the statistical enrichment (ie. over-representation) of the DEGs, we first conducted gene ontology (GO) term enrichment analysis using the Database for Annotation, Visualization and Integrated Discovery (DAVID) v6.7 functional enrichment tool[1]. This analysis, along with the subsequent pathway enrichment analysis, was performed separately for the up-regulated and down-regulated genes based on previous studies[2,3]. DAVID functional annotation clustering uses an algorithm to classify the similar, redundant and heterogeneous annotation contents from the same or different resources into annotation groups. We focused on GO FAT terms for biological processes (“GOTERM_BP_FAT”) so that too broad GO terms can be filtered out based on a measured specificity of each term. Only the GO terms with a conservative EASE score (a modified Fisher’s exact test p-value) less than 0.01 were considered significant. Clearly overlapping terms were collapsed into one term so that only the term with the lowest p value was retained.

DAVID was then used to identify over-represented pathways. Specifically, the DEGs uploaded to DAVID were mapped onto Biocarta[4], Kyoto Encyclopedia of Genes and Genomes (KEGG)[5], and Reactome[6] pathway databases, separately for the up-regulated and down-regulated genes.

To construct protein-protein interaction network and analyze its enrichment, the DEGs were submitted to the Search Tool for the Retrieval of Interacting Genes/Proteins (STRING) v9.1 software[7]. Finally, literature mining was performed to scrutinize the top 20 genes of the DEGs list in terms of their relevance to depression and central nervous system. To this end, we used Chilibot[8], a literature analysis software that identifies co-occurrence of names of a given gene (or genes) and a given keyword. The search term "depression" or "central nervous system" was used as the keyword. Each literature obtained by Chilibot was manually checked to ensure that the search term was used to describe what we meant (for example, literature on "long-term depression", a lasting change in synaptic transmission, was excluded). In addition, manual PubMed literature searches were conducted on the same 20 genes to check for other important evidence for their involvement in the brain.

References

1. Huang da, W., Sherman, B.T. & Lempicki, R.A. Systematic and integrative analysis of large gene lists using DAVID bioinformatics resources. *Nat. Protoc.* **4**, 44-57 (2008).
2. Hauck, S.M. *et al*. Deciphering membrane-associated molecular processes in target tissue of autoimmune uveitis by label-free quantitative mass spectrometry. *Mol. Cell Proteomics* **9**, 2292-2305 (2010).
3. Toker, L. *et al*. Inositol-related gene knockouts mimic lithium's effect on mitochondrial function. *Neuropsychopharmacology* **39**, 319-328 (2014).
4. Nishimura, D. BioCarta. *Biotech Software & Internet Report* **2**, 117-120 (2001).
5. Kanehisa, M. & Goto, S. KEGG: Kyoto encyclopedia of genes and genomes. *Nucleic Acids Res.* **28**, 27-30 (2000).
6. Joshi-Tope, G. *et al*. Reactome: a knowledgebase of biological pathways. *Nucleic Acids Res.* **33**, D428-432 (2005).
7. Franceschini, A. *et al*. STRING v9.1: protein-protein interaction networks, with increased coverage and integration. *Nucleic Acids Res.* **41**, D808-815 (2013).
8. Chen, H. & Sharp, B.M. Content-rich biological network constructed by mining PubMed abstracts. *BMC Bioinformatics* **5**, 147 (2004).
